# Effect of Transcranial Direct Current Stimulation versus Virtual Reality on Gait for Children with Bilateral Spastic Cerebral Palsy: A Randomized Clinical Trial

**DOI:** 10.3390/children10020222

**Published:** 2023-01-27

**Authors:** Asmaa Radwan, Hoda A. Eltalawy, Faten Hassan Abdelziem, Rebecca Macaluso, Megan K. O’Brien, Arun Jayaraman

**Affiliations:** 1 Physical Therapy Department for Women and Child Health, Faculty of Physical Therapy, Beni-Suef University, Beni Suef 62511, Egypt; 2 Department of Physical Therapy for Pediatrics, Faculty of Physical Therapy, Cairo University, Giza 12624, Egypt; 3 Department of Physical Therapy for Pediatrics, Faculty of Physical Therapy, October 6 University, 6th of October City 12511, Egypt; 4 Max Nader Lab for Rehabilitation Technologies and Outcomes Research, Shirley Ryan Ability Lab, Chicago, IL 60611, USA; 5 Department of Physical Medicine and Rehabilitation, Northwestern University, Chicago, IL 60611, USA

**Keywords:** transcranial direct current stimulation, virtual reality, gait intervention, bilateral spastic, cerebral palsy

## Abstract

Impaired gait is a common sequela in bilateral spastic cerebral palsy. We compared the effects of two novel research interventions—transcranial direct current stimulation and virtual reality—on spatiotemporal and kinetic gait impairments in children with bilateral spastic CP. Forty participants were randomized to receive either transcranial direct current stimulation or virtual reality training. Both groups received standard-of-care gait therapy during the assigned intervention and for the subsequent 10 weeks afterward. Spatiotemporal and kinetic gait parameters were evaluated at three different times: (i) before starting the intervention, (ii) after two weeks of intervention, and (iii) 10 weeks after intervention completion. Both groups exhibited higher velocity and cadence, as well as longer stance time, step length, and stride length after intervention (*p* < 0.001). Only the transcranial direct current stimulation group exhibited increased maximum force and maximum peak pressure after intervention (*p*’s ≤ 0.001), with continued improvements in spatiotemporal parameters at follow-up. The transcranial direct current stimulation group had higher gait velocities, stride length, and step length at follow-up compared to the virtual reality group (*p* ≤ 0.02). These findings suggest that transcranial direct current stimulation has a broader and longer-lasting effect on gait than virtual reality training for children with bilateral spastic cerebral palsy.

## 1. Introduction

Cerebral palsy (CP) is caused by early-stage brain injury, affecting 2 to 3 children in every 1000 live births. CP is defined as “a group of permanent disorders of the development of movement and posture causing activity limitation, that are attributed to non-progressive disturbances that occurred in the developing fetal or infant brain” [1]. CP is divided into different subtypes depending on the dominant neurological signs—spastic, dyskinetic, or ataxic [2]. Most the children with CP had spastic CP (77.4%), which can be divided into bilateral spastic CP (63.6%) and unilateral spastic CP (36.4%) [3]. CP is one of the most common developmental disabilities throughout life, caused by large-scale changes in subcortical brain activity with a reduced activation of corticospinal and somatosensory circuits, which leads to diminished activation of the central nervous system during volitional activities [4,5,6]. CP is accompanied by a host of medical comorbidities, each of which may have different strategies for optimal treatment and management [6,7].

Gait impairment is seen in 90% of children with bilateral spastic CP, stemming from this decreased cortical excitability and compounded by spasticity of the lower extremities, excessive muscular weakness, impaired joint mobility, and poor coordination and balance [8]. Specifically, children with CP have reduced gait velocity, cadence, and stride length, among other affected spatiotemporal gait parameters [9]. The International Classification of Functioning Disability and Health framework identifies gait patterns as measures of interest regarding overall health and disability [10]. This framework can be used globally in conjunction with impairment-based interventions to set goals related to reducing functional limitations of children with CP, such as by increasing activity, mobility, and participation (which are, by definition, limited in this population) [1,11]. Additionally, crouched gait, scissoring, and other atypical gait patterns are common in this population, further affecting the kinematic and kinetic characteristics of gait and leading to metabolically expensive locomotion, high fall risk, and long-term musculoskeletal injury [12]. For children with CP, the primary goal of rehabilitation is to facilitate mobility and appropriate walking patterns with or without external assistance. Improving spatiotemporal and kinetic characteristics of gait would improve gait function, increase gait efficiency, and reduce the risk of long-term disability. In turn, it would allow these children to participate in more activities of daily living, meaningful interactions with family and society, and environmental exploration, as well as to improve their physical development [13].

In the current study, we considered two technology-driven strategies that could potentially target gait impairments and improve gait function in children with CP: virtual reality (VR) and transcranial direct current stimulation (tDCS). Specifically, VR can simulate real-life activities while providing repetition, augmented sensory input and feedback, error reduction/augmentation, and gamification to increase motivation during the rehabilitation process [14,15]. Accordingly, there are three types of VR therapy—immersive, semi-immersive, and non-immersive [15]. As a training tool, VR provides visual-perceptual stimulation resulting from dynamic changes in context, which may aid in the execution of regulated exercises while also requiring concentration and additional postural control. Neuroimaging studies suggest that VR can facilitate learning and recovery by stimulating cortical reorganization and neural plasticity [16,17]. Previous research has utilized VR as a therapeutic tool for children to improve balance, walking speed, and/or distance, as well as to encourage physical activity [18]. Additional VR therapies have been shown to enhance functional performance in activities including squatting, standing posture, and energy expenditure [19,20]. With the commercialization of VR-related products such as the Nintendo Wii, many virtual games are readily available for home use. These games are often designed to challenge and train balance, posture, and dynamic movements, all of which are critical factors for gait. Thus, VR-based rehabilitation may offer a unique, accessible therapeutic approach to reduce gait impairments and improve dynamic function. 

In contrast, tDCS is a neuromodulation technique focused on optimizing existing neural pathways to prolong and/or improve the functional gains achieved by rehabilitation [4]. tDCS is applied through either anodal or cathodal stimulation, which corresponds to excitation or inhibition of the stimulated brain areas, respectively. Anodal stimulation enhances cortical excitability through depolarization, allowing for more spontaneous cell firing, while cathodal stimulation has an inhibitory effect through hyperpolarization. Functionally, this means application of tDCS will influence activity in the area of the brain it targets. Previous research indicates that inhibited cortical input to the corticospinal tract is a possible cause of increased spasticity in CP, so it is reasonable to predict that anodal stimulation would mitigate these symptoms in individuals with spastic CP [21,22]. The neurophysiological effects of anodal tDCS can also potentiate motor learning through this increase in cortical activity, which is applicable to the treatment of all subtypes of CP. These benefits may translate into functionally improved gait as well [21]. 

While both tDCS and VR have been found to amplify the positive effects of training, further investigation is required to determine their clinical effectiveness for children with CP as recommended by the previous literature. Both methods have a positive evidence base to support their use as motor interventions, though the evidence base is still moderate-to-weak, suggesting additional studies are needed [6,15]. For tDCS, there are also limited data for the pediatric population, with more research to date in adults than children [6,21,22]. Thus, the aim of this study was to directly compare the effects of tDCS and VR training on spatiotemporal and kinetic gait parameters in children with bilateral spastic CP, as a supplemental intervention to traditional physical therapy. We hypothesized that there would be a similar effect between tDCS and VR on gait patterns in children with bilateral spastic CP.

## 2. Materials and Methods

### 2.1. Participants

**Figure 1:**
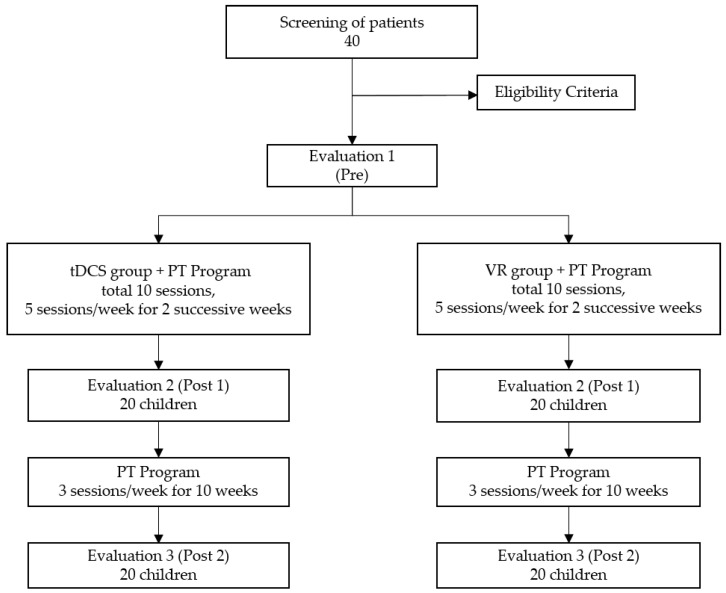
Flowchart of study based on consolidated standards of reporting trials (CONSORT).

The study flow for this prospective randomized clinical trial is presented in Figure 1. The randomization was generated by block randomization. A convenience sample of 40 children (23 male, 17 female) were recruited from a specialized children’s outpatient clinic run by the faculty of physical therapy at Cairo University (Egypt). The inclusion criteria were the following: diagnosed with bilateral spastic CP and between the ages 7–12 years old; minimum spasticity grades of 1 and 1+ according to the modified Ashworth Scale; and gross motor function classification system (GMFCS) at level I or II. The age range was chosen to make sure the gait is matured and any changes were due to interventions rather than ongoing motor development [23]. GMFCS level was chosen to ensure that children could participate in both virtual reality and gait training [21]. Exclusion criteria included the following: children who had visual impairments, hearing damage, history of epilepsy or seizures, fixed deformities at lower limbs, history of orthopedic surgeries, or injection with botulinum toxin in the previous year; metal implants in the skull, or inability to understand the task. Prior to participation, the parents or legal guardians of the children provided verbal consent to participate, as approved by the ethical committee of the Faculty of Physical Therapy at Cairo University.

### 2.2. Experimental Setup 

Children were randomly assigned to one of two groups receiving either tDCS or VR training as a research intervention for two weeks. Gait assessments were performed at three different times—prior to the start of the intervention (Pre), after completing two weeks of the intervention (Post 1), and after 10 additional weeks of standard-of-care physical therapy (Post 2). The PT program was added to the study for two reasons. One, given that gait metrics were the primary outcome, it was clinically reasonable to combine additional gait training with the research interventions. This would be the likely scenario for real-world clinical implementation, to administer these interventions alongside standard-of-care, gait-centered PT. Second, the ethical committee in Egypt supported including PT in the study given the experimental nature of the two interventions, to maximize the possibility that children participating in research would benefit from the study given their time and effort.

### 2.3. Gait Assessments

Spatiotemporal and kinetic gait parameters were measured at the three assessment times using Walkway^TM^ Pressure Measurement System (Tekscan, Inc.; South Boston, MA, USA). This system consists of a digital mat (195.5 cm length, 44.2 width) inserted in a wooden walkway, equipped with sensors (4 sensels/cm^2^) and a pressure recording system (1 to 862 kPa) at a sampling resolution up to 185 Hz. A computer with the Tekscan Software (version 7; Tekscan, Inc.; South Boston, MA, USA) was used to download the data. 

A calibration procedure was carried out prior to any step recordings by asking the participant to stand for 5 to 6 s on each leg. They were then asked to walk the length of the mat. All children were given a trial test to become comfortable with the walkway and the setting. Three trials were then completed to collect the gait parameters for analysis. Spatiotemporal parameters included velocity, cadence, step length, stride length, stance time, and swing time, while kinetic parameters included maximum force and maximum peak pressure.

### 2.4. Intervention Procedures

#### 2.4.1. tDCS Group

**Figure 2:**
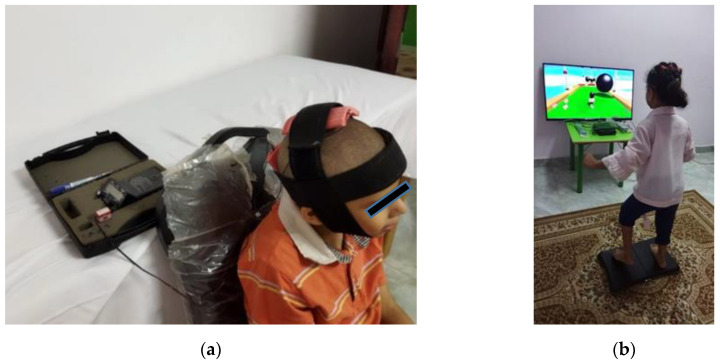
Experimental setup for (**a**) tCDS, including electrode position; (**b**) VR balance training, including an example obstacle course game.

Children assigned to the tDCS group received active tCDS at their primary motor cortex (ActivaDose II, serial no. 13070350; ActivaTek, Inc.; Gilroy, CA, USA). Stimulation was conducted at an intensity of 1 mA for 20 min per session, 5 times/week for 2 successive weeks (total of 10 sessions). An intensity of 1 mA was shown to be appropriate in children’s investigations [4,14]. The anode (+) was positioned on the midline sagittal plane of the skull (Cz), corresponding to the motor area of lower limbs, and the cathode (−) was positioned over the inion, as shown in Figure 2a.

#### 2.4.2. VR Group 

Children assigned to the VR group received virtual balance training using Nintendo Wii and Wii Balance Board, with a custom training program developed from activities on the Wii Fit Plus game. Training was conducted for 30 min, 5 sessions/week for 2 successive weeks (total of 10 sessions). Two sessions with Wii Fit Plus were conducted before the treatment protocol to help the children familiarize with the VR setup [24]. The VR setup, with an example game from the Wii Fit Plus, is shown in Figure 2b.

#### 2.4.3. PT Program

Both groups received the standard-of-care gait training in addition to their assigned intervention. During the two-week intervention phase, gait training was administered immediately after each intervention session. Training was delivered in one hour increments 5 times/week for those first two weeks, then 3 times/week for the next 10 weeks. The program included various gait training and balance tasks as well as resistive exercises and passive stretching as necessary. Task-specific gait exercises included the following: walking in a closed indoor environment using a separator or a stepper (forward, backward, and sideways), walking in an open indoor environment with obstacles (rolls or wedges of various sizes and heights), walking on various floor surfaces (spongy, hard, and on a mat), and climbing stairs up and down without assistance. The children also performed dynamic balance exercises by completing forward and sideways walking on a balance board. A stepper (raised agility ladder) was placed on the balance board to increase difficulty, requiring more concentration and motor control. Resistive exercises were applied for anti-spastic muscle groups. If needed, passive stretching was performed for all tight muscle groups. To ensure that the children were safe, the physiotherapist (A.R.) was on close supervision. 

### 2.5. Adverse Events 

All study participants and their caregivers were asked about possible side effects during each intervention session and after the completion of the protocol. During the sessions, participants in the tDCS group reported a tingling sensation at the beginning of stimulation, but this sensation either ceased after a few seconds or was not considered bothersome. Some subjects perceived a slight itching sensation at the site of stimulation. Four children in the tDCS group experienced mild headaches. No other side effects or significant adverse events were reported.

### 2.6. Data Analysis

Statistical analyses were conducted using R version 4.2.2 (2019 R Foundation for Statistical Computing; Vienna, Austria). All variables were tested for normality using the Shapiro–Wilk test. The homogeneity between intervention groups was tested using Levene’s test, and a chi-squared test was used to compare sex, spasticity grades, and GMFCS levels between groups. Unpaired t-tests were conducted to compare other demographic characteristics between groups. Paired t-tests were conducted to compare gait parameters for the right and left legs and parameters were averaged between legs if there was no significant difference between them. Outliers, defined as values more than three standard deviations from the group mean, were removed prior to analysis. A linear mixed model was created for each gait parameter with time and group as fixed factors and subject as a random factor. A two-way ANOVA was applied to the model to compare effects of time, intervention, and their interaction. If significance was observed, Tukey’s honestly significant difference test was used to evaluate pairwise significance (*emmeans* package in R). The level of significance for all statistical tests was set to 0.05.

## 3. Results

Demographics and clinical characteristics of the two intervention groups were consistent and homogenous, with no statistically significant differences between them (Table 1). Between-group and within-group comparisons of gait parameters are provided in Table 2, Table 3 and Table 4 and summarized below. We found there were no significant differences in gait parameters between the right and left legs for either group in any gait parameter (*P*’s > 0.06); therefore, we combined data from bilateral parameters (e.g., right step length, left step length) into single parameters (e.g., step length).

### 3.1. Effects of Time and Intervention Group

Results from the two-way ANOVA applied to the linear mixed models are summarized in Table 2. Time as a factor had a significant effect on all parameters except swing time (F (2.76) = 1.15, *p* = 0.32). Intervention group as a factor had a significant effect on gait velocity only (F(1.38) = 4.35, *p* = 0.044). Since many parameters showed a significant effect in the interaction between time and group, pairwise comparisons were also evaluated.

### 3.2. Temporal Parameters

**Figure 3:**
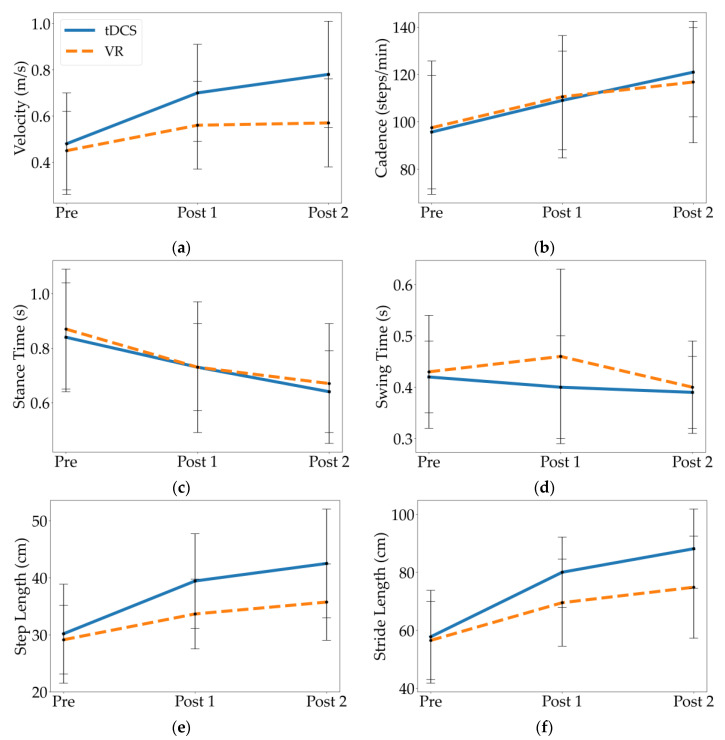
Mean and standard deviation of spatiotemporal parameters at Pre, Post 1, and Post 2 for the tDCS and VR groups, including (**a**) velocity, (**b**) cadence, (**c**) stance time, (**d**) swing time, (**e**) step length, and (**f**) stride length.

When comparing Pre to Post 1 and Pre to Post 2, both the tDCS and VR groups had statistically significant differences in most temporal parameters, exhibiting higher gait velocity and cadence, and longer stance time relative to their baseline (*p*’s ≤ 0.01; Table 3, Figure 3). When comparing Post 1 to Post 2, only the tDCS group had significant differences in these same parameters after the 10 additional weeks of PT (*p*’s ≤ 0.01). There were no changes in swing time at any time-point within the groups. Additionally, the tDCS group demonstrated a significantly higher gait velocity than the VR group at Post 1 and Post 2 (*p*’s ≤ 0.03; Figure 3). There were no significant differences between the groups for cadence, stance time, or swing time.

### 3.3. Kinetic Parameters

**Figure 4:**
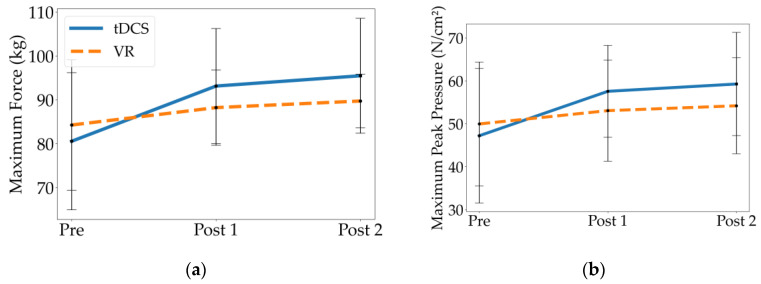
Mean and standard deviation of kinetic parameters at Pre, Post 1, and Post 2 for the tDCS and VR groups, including (**a**) maximum force, and (**b**) maximum peak pressure.

When comparing Pre to Post 1 and Pre to Post 2, only the tDCS group had statistically significant differences in kinetic parameters, exhibiting greater maximum force and maximum peak pressure relative to their baseline (*p*’s ≤ 0.001; Table 4, Figure 4). The VR group showed no significant differences between time-points for these kinetic parameters. There were also no significant differences between the groups in maximum force or maximum peak pressure. When comparing Post 1 to Post 2, neither group had significant differences in their kinetic parameters.

## 4. Discussion

This study compared the effects of tDCS and VR training on gait metrics when combined with standard gait training in children with bilateral spastic CP. Both interventions improved spatiotemporal gait parameters following treatment, including gait velocity, cadence, stance time, step length, and stride length. While both groups demonstrated additional gains in step length and stride length 10 weeks after treatment (during continued gait training), only children who received tDCS demonstrated additional temporal gains (i.e., velocity, cadence, stance time). Additionally, children who received tDCS demonstrated significantly higher gait velocities, step lengths, and stride lengths after completing the two-week intervention as well as at the 10-week follow-up than those who received VR training. Only tDCS improved the maximum force and peak pressure following two weeks of intervention, and there were no significant differences between the intervention groups for these kinetic parameters.

Gait velocity has an important relationship with the cadence. In this study, children who received tDCS demonstrated higher gait velocities at follow-up than children who received VR training, though there was no significant difference in cadence between the two groups at this time-point. This outcome is likely explained by differences in step and stride length [25], since the tDCS group also had significantly longer step/stride lengths than the VR group at follow-up (Table 3; Post 2). Potentially, this may be due to the modulating effect of tDCS on the primary motor cortex, contributing to improved voluntary motor control. Thus, active tDCS seems to have potentiated the effects of motor training, as the children in this group demonstrated a better performance regarding activities that require functional control of the body [14,25]. Moreover, gait velocity is a variable that has been associated with independence and fall reduction, as walking difficulties may severely limit children’s daily activities and gait speed may be predictive of their level of community ambulation. This finding may indicate that tDCS application has the potential to improve the level of community ambulation in children with CP [21].

To our knowledge, no previous research has compared the separate effects of a VR training program and tDCS. Instead, most studies in this area have investigated therapies that incorporate both VR and tDCS, or that have examined only one of these interventions without comparison to the other. In the present study, both groups improved spatiotemporal parameters, but only the tDCS group exhibited changes in the kinetic gait over two weeks of delivery with supplemental standard-of-care gait training, and these gains were maintained or further improved over an additional 10 weeks of gait training. Although we cannot disentangle the impact of each intervention from the supplemental gait training, and it is likely that this training contributed to the improvement and maintenance of spatiotemporal/kinetic parameters, it is important to note that neither of the experimental interventions delivered additional gait practice beyond the standard of care. tDCS involved stimulation while sitting, and VR training was geared toward balance tasks. Thus, it is interesting to see differences between the intervention groups, which are likely attributable to the interventions themselves. While both groups had similar gait characteristics at baseline (Pre), and both improved over the entire 12 weeks (Pre to Post 2), the tDCS group exhibited larger gains than the VR group concerning gait velocity, step length, and stride length at the follow up visit. The results of the current study show that VR did not have any impact on the kinetic parameters, however it might have had an impact if the VR program had lasted longer than two weeks. Since neither intervention had a significant impact on swing time, the changes in gait velocity and cadence may be attributed predominantly to decreases in stance time.

Our findings are in alignment with previous tDCS research. A study by Grecco et al. [26] performed a similar intervention for children with bilateral spastic CP, applying tDCS over the primary motor cortex for 10 sessions (5 sessions/week for two weeks) during treadmill training. They found that active tDCS improved gait velocity (mean difference: 0.3 m/s, compared to 0.22 m/s in our study from Pre to Post 1) and cadence (mean difference: 19.5 steps/min, compared to 13.37 steps/min in our study from Pre to Post 1). These results were maintained one month after the end of the intervention. No significant differences in gait variables were found in a control group (treadmill training + placebo tDCS), supporting the idea that administration of tDCS potentiates the effects of motor training.

Another study by Grecco et al. [25] found that tDCS applied to the primary motor cortex resulted in positive changes in gait and static balance for children with CP, whereas a control group receiving sham tDCS exhibited no changes. Only one session of tDCS was needed to significantly increase step length and gait velocity. Grecco et al. [27] also found that VR gait training combined with anodal tDCS at the primary motor cortex had a positive effect on temporal gait variables (velocity and cadence), gross motor function, and motor ability in children with CP at post-treatment assessment, with gains maintained at one month following the intervention. This approach led to a significant change in motor cortex plasticity, as evidenced by an increased motor excitation potential amplitude. Similarly, Kaski et al. [28] also found that applying 15-min anodal tDCS stimulation to the prefrontal or primary motor cortex before walking on a motion platform improved ability postural control and gait velocity compared to sham tDCS. These results demonstrated that tDCS induced changes in motor cortex excitability, promoting motor control and lower extremity movements, resulting in enhanced locomotor control with improved gait [24].

Interestingly, previous work with VR training also supports VR as a way to cause neuroplastic changes in the sensory motor cortex and improve motor performance of affected limbs. On functional MRI, enlargement of the primary motor area on the side of the body that is contralateral to the affected limb and increased activity of the cerebellum was seen after training with VR [18]. There have been several recent studies also examining the effect of VR training on gait parameters. For example, Biffi et al. [29] compared robot-assisted gait therapy (RAGT) to virtual reality treadmill training (VRTT), and both were coupled with standard physiotherapy. Although the VRTT was not more effective than RAGT in improving step length and stride length, the gait deviation index had a statistically significant improvement only in the VRTT group. Valenzuela et al. [30] also evaluated the effects of a short-term intensive VR intervention on gait speed in four adolescents with different types of CP. A two-week intervention phase was performed with 12 Nintendo^®^ Wii games in six sessions (90 min) per week. The results showed trends in improved gait speed, near 2 SDs above baseline (at least ~0.1 m/s from visual inspection for adolescents with bilateral CP, compared to 0.11 m/s for the VR group in our study from Pre to Post 1). Beyond gait, Jha et al. [31] conducted a study to see how VR and PT affected balance, gross motor skills, and daily functioning among children with bilateral spastic CP. Combined VR and PT showed substantial improvement only in the Kids-Mini-Balance Evaluation System Test post training and during the 2-months follow-up opposed to PT alone.

In our current study, even though both research interventions showed significant within-group improvements over time, tDCS showed greater improvements in most gait metrics between groups and showed continued gains in both spatial and temporal gait parameters for 10 weeks, while the VR group showed continued gains in spatial, but not temporal, parameters. These results strongly suggest that tDCS in combination with gait training is an effective neuromodulatory intervention to reduce gait impairments and improve function in children with bilateral spastic CP. 

## 5. Limitations

Additional studies will be needed to directly compare the neurophysiological effects of tDCS and VR, such as by using electroencephalography (EEG) or TMS to measure changes in cortical activity and motor excitability. Our study did not include a control group (i.e., children receiving no intervention but still completing the standard of care gait training), since our objective during this initial study was to compare the tDCS and VR interventions and their relative efficacy as a supplement to a standard gait training program [32]. Combining these interventions with standard gait training reflects how they would likely be implemented in real-world clinical practice. However, other studies have begun to combine these interventions together [33] or with hydrotherapy [34] or neurodevelopmental therapy (NDT) [35]. Future work will extend this study into a randomized control trial to determine the specific impact of each intervention, or their combination, compared to gait training.

## 6. Conclusions

When combined with standard gait training, both tDCS and VR yielded positive improvements in spatiotemporal gait parameters after two weeks of intervention. Only tDCS yielded improvements in kinetic gait parameters. tDCS produced higher gait velocities, stride lengths, and step lengths at a 10-week follow-up in comparison with the VR. Taken together, these findings suggest that tDCS produced a broader and longer-lasting impact on gait, facilitating a carryover effect when children continued with standard PT alone.

## Figures and Tables

**Table 1:** Demographics and clinical characteristics of study participants, with between-group comparison.

		tDCS	VR	MD	t-Value	*p*-Value	Sig
		Mean ± SD	Mean ± SD				
Age (years)		8.6 ± 1.56	8.45 ± 1.53	0.15	0.31	0.76	NS
Weight (kg)		25.61 ± 8.44	25.84 ± 7.94	−0.23	−0.09	0.92	NS
Height (cm)		126.95 ± 11.58	124.25 ± 9.62	2.7	0.8	0.42	NS
BMI (kg/m^2^)		15.95 ± 4.82	16.39 ± 2.81	−0.44	−0.35	0.72	NS
Spasticity	Grade I	16 (80%)	14 (70%)	χ^2^ 0.53		0.46	NS
	Grade I+	4 (20%)	6 (30%)				
GMFCS	Level I	14 (70%)	12 (60%)	χ^2^ 0.44		0.37	NS
	Level II	6 (30%)	8 (40%)				

SD: standard deviation; MD: mean difference between groups; t-value: unpaired t-value; *p*-value: probability value; χ^2^: chi-squared value; Sig: between-group significance; NS: non-significant.

**Table 2:** Results from ANOVA applied to linear mixed models.

Parameter	Time		Group		Time × Group	
	F	*p*-Value	F	*p*-Value	F	*p*-Value
Velocity	157.04	<0.001 *	4.35	0.044 *	25.95	<0.001 *
Cadence	52.88	<0.001 *	0.0014	0.97	1.20	0.31
Stance Time	39.30	<0.001 *	0.16	0.70	0.40	0.67
Swing Time	1.15	0.32	1.00	0.32	0.25	0.78
Step Length	150.21	<0.001 *	3.76	0.060	14.67	<0.001 *
Stride Length	139.04	<0.001 *	3.71	0.061	8.74	<0.001 *
Maximum Force	19.05	<0.001 *	0.26	0.61	8.15	<0.001 *
Maximum Peak pressure	16.25	<0.001 *	0.39	0.53	4.09	0.021 *

F: F-statistic; *p*-value: Probability value; * *p <* 0.05.

**Table 3:** Between-group and within-group comparisons of spatiotemporal parameters at Pre, Post 1, and Post 2.

	Between Group			Within Group		
Spatiotemporal Parameters	Pre	Post 1	Post 2	*p*-Value		
	Mean ± SD	Mean ± SD	Mean ± SD	Pre vs. Post 1	Pre vs. Post 2	Post 1 vs. Post 2
Velocity (m/s)						
tDCS	0.48 ± 0.22	0.70 ± 0.21	0.78 ± 0.23	<0.001 *	<0.001 *	<0.001 *
VR	0.45 ± 0.17	0.56 ± 0.19	0.57 ± 0.19	<0.001 *	<0.001 *	0.54
	*p* = 0.61	*p* = 0.03 *	*p* = 0.02 *			
Cadence (steps/min)						
tDCS	95.67 ± 23.97	109.04 ± 20.81	120.95 ± 18.85	0.001 *	<0.001 *	<0.001 *
VR	97.50 ± 28.16	110.58 ± 25.84	116.78 ± 25.67	<0.001 *	<0.001 *	0.12
	*p* = 0.81	*p* = 0.84	*p* = 0.59			
Stance time (s)						
tDCS	0.84 ± 0.20	0.73 ± 0.16	0.64 ± 0.15	0.01 *	<0.001 *	0.01 *
VR	0.87 ± 0.22	0.73 ± 0.24	0.67 ± 0.22	<0.001 *	<0.001 *	0.14
	*p* = 0.57	*p* = 0.99	*p* = 0.61			
Swing time (s)						
tDCS	0.42 ± 0.07	0.40 ± 0.10	0.39 ± 0.07	0.78	0.46	0.86
VR	0.43 ±0.11	0.46 ± 0.17	0.40 ± 0.09	0.94	0.70	0.50
	*p* = 0.78	*p* = 0.25	*p* = 0.54			
Step length (cm)						
tDCS	30.18 ± 8.68	39.43 ± 8.29	42.50 ± 9.53	<0.001 *	<0.001 *	<0.001 *
VR	29.13 ± 6.03	33.65 ± 6.13	35.73 ± 6.70	<0.001 *	<0.001 *	0.03 *
	*p* = 0.67	*p* = 0.02 *	*p* = 0.01 *			
Stride length (cm)						
tDCS	57.76 ± 16.03	80.04 ± 12.15	88.15 ± 13.67	<0.001 *	<0.001 *	<0.001 *
VR	56.52 ± 13.47	69.53 ± 15.02	74.86 ± 17.60	<0.001 *	<0.001 *	0.04 *
	*p* = 0.79	*p* = 0.03 *	*p* = 0.01 *			

SD: Standard deviation; * *p* < 0.05.

**Table 4:** Between-group and within-group comparisons of kinetic parameters at Pre, Post 1, and Post 2.

	Between Group			Within Group		
Kinetic Parameters	Pre	Post 1	Post 2	*p*-Value		
	Mean ± SD	Mean ± SD	Mean ± SD	Pre vs. Post 1	Pre vs. Post 2	Post 1 vs. Post 2
Maximum force (kg)						
tDCS	80.56 ± 15.64	93.14 ± 13.10	95.48 ± 13.09	<0.001 *	<0.001 *	0.54
VR	84.26 ± 14.86	88.23 ± 8.58	89.73 ± 6.13	0.64	0.27	0.78
	*p* = 0.14	*p* = 0.19	*p* = 0.13			
Maximum peak pressure (N/cm^2^)						
tDCS	47.20 ± 15.67	57.55 ± 10.70	59.25 ± 12.02	<0.001 *	0.001 *	0.71
VR	49.95 ± 14.41	53.05 ± 11.80	54.18 ± 11.18	0.33	0.13	0.86
	*p* = 0.50	*p* = 0.27	*p* = 0.21			

SD: Standard deviation; * *p* < 0.05.

## Data Availability

The data that supports the findings of this study is available from the corresponding author upon reasonable request and approval from Cairo University.
